# Short-term activity cycles impede information transmission in ant colonies

**DOI:** 10.1371/journal.pcbi.1005527

**Published:** 2017-05-10

**Authors:** Thomas O. Richardson, Jonas I. Liechti, Nathalie Stroeymeyt, Sebastian Bonhoeffer, Laurent Keller

**Affiliations:** 1 Department of Ecology and Evolution, University of Lausanne, Switzerland; 2 Department of Environmental Systems Science, ETH Zürich, Switzerland; Northeastern University, UNITED STATES

## Abstract

Rhythmical activity patterns are ubiquitous in nature. We study an oscillatory biological system: collective activity cycles in ant colonies. Ant colonies have become model systems for research on biological networks because the interactions between the component parts are visible to the naked eye, and because the time-ordered contact network formed by these interactions serves as the substrate for the distribution of information and other resources throughout the colony. To understand how the collective activity cycles influence the contact network transport properties, we used an automated tracking system to record the movement of all the individuals within nine different ant colonies. From these trajectories we extracted over two million ant-to-ant interactions. Time-series analysis of the temporal fluctuations of the overall colony interaction and movement rates revealed that both the period and amplitude of the activity cycles exhibit a diurnal cycle, in which daytime cycles are faster and of greater amplitude than night cycles. Using epidemiology-derived models of transmission over networks, we compared the transmission properties of the observed periodic contact networks with those of synthetic aperiodic networks. These simulations revealed that contrary to some predictions, regularly-oscillating contact networks should impede information transmission. Further, we provide a mechanistic explanation for this effect, and present evidence in support of it.

## Author summary

Many complex biological systems, from cardiac tissues to entire animal populations, exhibit rhythmical oscillations. Here we studied a textbook example of a complex living system–colonies of *Leptothorax* ants, which exhibit short (15 minute) collective activity cycles. In ant colonies, information, food, and chemical signals are transported throughout the group via worker-to-worker physical contacts, and it has therefore been suggested that the activity cycles might serve to increase the rapidity of information transmission. To test this, we used an automatic ant tracking system to identify physical contacts between workers, from which we reconstructed the dynamical network of physical contacts. We used models of information transmission derived from the study of contagious diseases to simulate information transmission over the rhythmical contact networks, which we compared against a set of comparable networks that exhibited no rhythms. These comparisons showed that, contrary to the expectations, oscillatory activity cycles slowed down information transmission rather than speeding it up. We suggest that the colony activity cycles might serve to ensure that old or out-of-date information is quickly expunged, and potentially reduce interference between different information streams.

## Introduction

Cyclical activity patterns are found at every level of biological organization, from genes to cells, organs, and societies, and span many orders of magnitude in space and time. For instance, cyclical activity is found at the smallest spatial scale in the regulation of circadian *Clock* genes [1], and at the largest, in the cyclical fluctuation of populations of predators and prey [2, 3]. Similarly, cyclical phenomena occur at frequencies ranging from roughly once per second for the firing patterns of human cardiac pacemaker cells [4], to once every 13 or 17 years for reproductive cycles in cicadas [5].

Although cyclical activity patterns can be driven by an exogenous signal, such as diurnal, lunar and seasonal cycles, there are also many systems in which cyclical activity emerges in the absence of a pacemaker. For example, in humans an applauding audience may spontaneously break into bouts of synchronized clapping [6]. Similarly, the synchronization (or anti-synchronization) of courting fireflies [7] and calling in frog choruses [8] is an emergent property, though these are so regular that it was originally hypothesized that there must exist a leader that sets the rhythm [9, 10].

**Fig 1:**
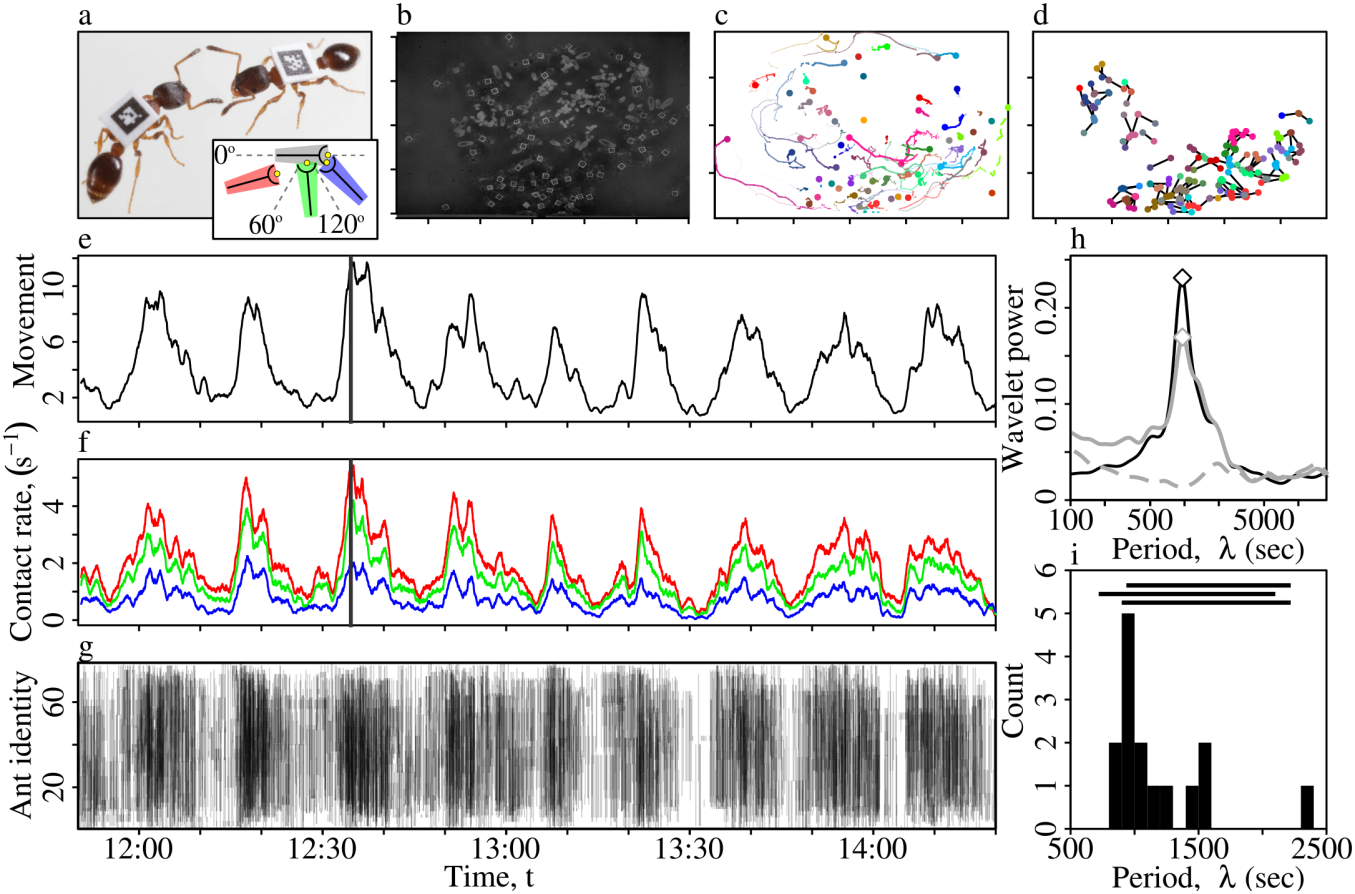
Short-term activity cycles. (**a**)*Leptothorax acervorum* workers with unique ARtag markers glued to the thorax. The inset illustrates the geometrical approximation used to infer ant-to-ant contacts. Blue trapezoid: A head-to-head contact with the grey ant is detected as the angle between them is high (*θ*≥120°), and the interaction point (yellow circle) of the blue ant enters into the trapezoid of the grey. Green trapezoid: When the angular difference threshold is relaxed (*θ*≥60°), both head-to-body and head-to-head contacts are detected. Red trapezoid: No contact is detected as the ants are too far apart. (**b**) Image of the nest during a period of high activity (colony 13, replicate 1, 12:34 p.m., indicated by the vertical line in panel e). The triangle indicates the entrance. The axis tick-marks denote 1cm units. Panels c-d summarise the spatial distribution of the activity occurring during 1 minute preceding the midday activity peak (12:33–12:34 p.m.). (**c**) Ant spatial trajectories. Each ant is arbitrarily assigned a different colour. (**d**) Contacts between ants. Black lines indicate contacts between ants. Colours correspond to those in panel c. Panels e-g show a 2.5 hour segment from the same recording session. The colony exhibits short-term periodic oscillations in (**e**) the overall movement activity, (**f**) the overall contact rate, and (**g**) in the time-ordered contact network. Different coloured lines in (**f**) represent different contact definitions. Black: inclusive definition, where all contacts are counted irrespective of the angular difference between the two participants, *θ*≥0°. Blue: the conservative definition that considers only head-on contacts, *θ*≥120°. Green: intermediate definition, *θ*≥60°. In (**g**) the black vertical lines represent the start of a physical contact between two ants (contact duration omitted for clarity). (**h**) Wavelet power spectra for the full 3-day observation period, for colony 13, replicate 1. Solid black line: Wavelet periodogram for the movement activity. Solid grey line: Periodogram for the contact rate (for *θ*≤120°). Dashed grey line: Periodogram for contact rate in the aperiodic synthetic networks, produced by the null model. Diamonds indicate the dominant period, *λ*. Note, the x-axis is on a logarithmic scale. See the Supporting Information for the spectra for the 14 other replicates. (**i**) Distribution of the dominant periods *λ* for the contact rate time-series, across all 15 three-day time-series. Both the movement activity and contact rate time-series had a dominant period of *λ* = 960 seconds. The horizontal bars indicate previous estimates for the period length. Upper bar: 15.6-37 min range for *L. acervorum* [11]. Middle bar: 12-35 min range for *L. allardycei* [12] Lower bar: 15-37 min range for *L. allardycei* [12].

In this paper we study short-term activity cycles (henceforth, STACs) in colonies of the ant *Leptothorax acervorum* (Fig 1a). These ants display a remarkable degree of synchronization in their activity patterns, resulting in quasi-periodic oscillations in the overall rate of physical contacts between nestmates (S1 Video), with roughly three to four peaks per hour [11, 12, 13]. In colonies of social insects—ants, bees, wasps & termites—life within the nest is dominated by frequent physical contact between nestmates, such as allogrooming, and oral fluid exchange. This contact-based ‘infrastructure’ serves as a decentralized communication network for the transport of a wide variety of information-bearing materials. These include complex mixtures of cuticular hydrocarbons that are crucial for nestmate-recognition [14, 15, 16] and task-allocation [17], chemical fertility-signals advertising the presence of the queen [18, 19, 20], and growth hormones controlling the development of the brood [21]. Furthermore, the contacts themselves can convey information, without chemical transfer. For example, information transmission during cooperative foraging involves both ritualized tactile motor displays [22, 23, 24], and also purely passive (i.e. kinetic) encounters [25]. Given that physical contacts provide a substrate for communication, it follows that the speed of information transfer should depend upon the contact rate. Indeed, [26] first predicted that when activity is synchronized, information should spread more rapidly (see also [11, 13]). Although there are many examples of complex networks—both naturally-occurring, and human designed—that function to enhance transmission of information and other resources [27, 28], there are also examples in which the network structure functions to impede transmission of harmful materials such as pathogens [29] or poisons [30]. Therefore, in what follows we use a combination of experiment and simulation modelling to test if and how STACs influence transmission in ant colonies.

To test whether STACs could potentially influence information transmission, we used an automated tracking system [31] in which a unique barcode tag is attached to the thorax of every ant in the colony (Fig 1a and 1b), to acquire 15 three-day-long records of all the physical contacts occurring within the nests of *L. acervorum* ants. To characterise the transmission properties of these contact sequences, we use a modelling approach based on models of disease contagion in epidemiology [32, 33]. In addition to simulating disease spread, such models have been used to simulate information transmission over a range of human interaction networks, such as email and mobile phone call records, and face-to-face conversation logs [34, 35, 36], as well as over social insect contact networks [31, 37, 38]. Most previous analyses of the transmission properties of animal social networks have aggregated or summed repeated interactions between individuals, to obtain a static network representation [37, 39, 40]. However, because the presence of temporal clustering or intermittency can strongly influence the overall transmission properties [41, 42], we perform no such aggregation and instead consider the full time-ordered network formed by the raw contact sequence. By applying our epidemiology-derived simulation model of information transmission to the underlying time-respecting transmission pathways, we are able to investigate the influence of colony activity cycles on transmission properties, which would be impossible with a time-aggregated network representation. Then, by comparing the observed transmisison properties with the transmission properties of synthetic networks that do not exhibit periodicity, we show that STACs should impede transmission, not enhance it. Finally, we give a mechanistic explanation for this effect, and provide evidence that supports it.

## Results

### A two-state model of information transmission

**Fig 2:**
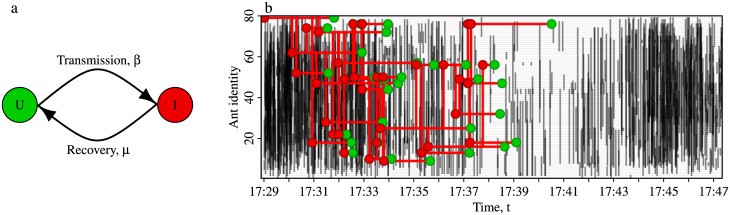
State transitions in the Uninformed-Informed-Uninformed (UIU) model of information transmission. (**a**) State transitions in the UIU model. Contacts between an informed ant (**I**), and an uninformed ant (**U**), can potentially result in information transmission from the former to the latter, in which case the uninformed ant becomes informed (U→I) at rate *β*. Informed ants spontaneously switch back to being uninformed at rate *μ* (I→U). (**b**) Simulated information transmission occurring over a ∼20 minute contact sequence (colony 6, replicate 1, afternoon of day 2). Black vertical lines represent the start of head-to-body physical contacts between ant pairs (conservative contact definition, *θ*≥120°). For clarity, contact durations are omitted from the plot. Red points represent transmission U→I transitions, green points represent I→U transitions. Here, a UIU simulation (*β* = 0.5, *μ* = 0.01), is initiated in the middle of an activity peak by infecting a single ant (at *t*_0_ = 17:29), but although several other ants do become informed, the oncoming quiescent period ensures that by t = 17:41 the information has been completely lost.

**Fig 3:**
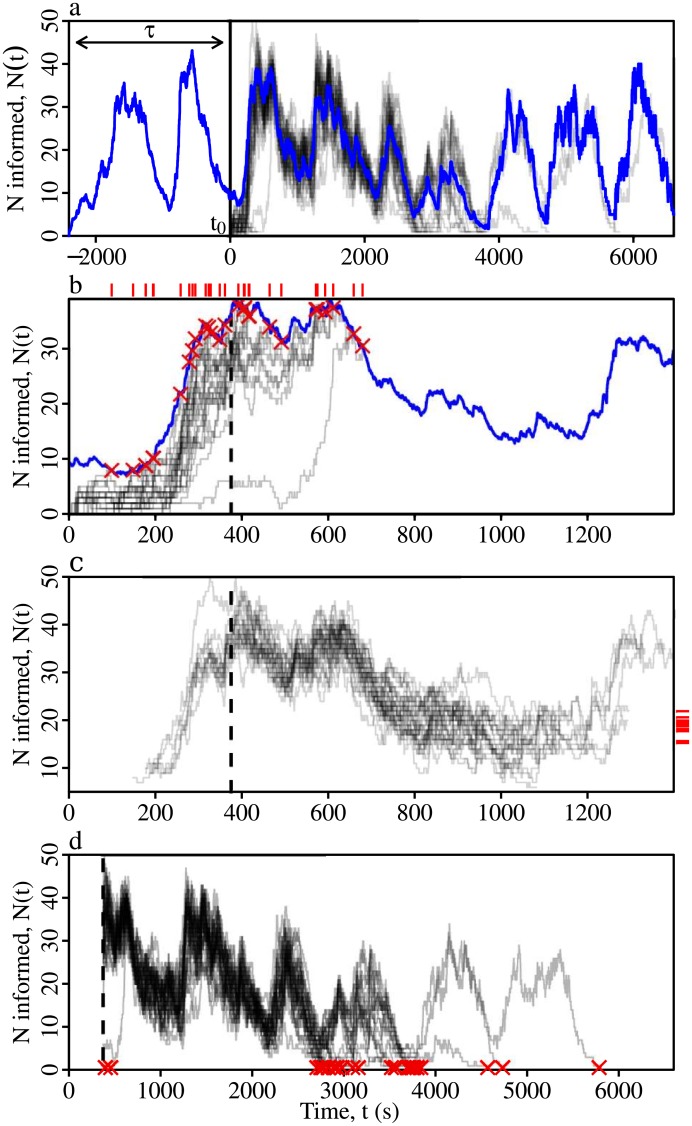
Assessing network transmission properties. (**a**) Defining the time-explicit steady state. Blue line—the ‘reference’ simulation ensemble, showing the mean number of informed ants $\bar{N_{t_{0}-\tau }\left(t\right)}$ across all extant simulations initiated at *t*_0_−*τ*. Grey lines—100 ‘focal’ runs, initiated at the start time, *t*_0_. To be defined as having reached the steady state, a focal run must reach the blue line. The delay between the reference and the focal simulation ensembles *τ*, was independently determined for each network and for each {*β*, *μ*} parameter combination. (**b**) Calculating the probability that the information becomes self-sustaining *P*_*sustain*_, is defined as the proportion of focal runs that reach the steady state (grey lines). Hitting times are indicated by the red crosses. The upper rugplot indicates the hitting time distribution, with the dashed vertical line indicating its mean $\bar{T}$, which marks the end of the growth phase. (**c**) Calculating the probability that an ant is informed, given that the information has become self-sustaining within the colony (i.e. the prevalence), *P*_*informed*_. Grey lines; focal runs that reach the steady state and that remain extant for at least 1 hour thereafter (here we use 6 minutes), are used to calculate the information prevalence. The right rugplot shows the distribution of the mean number of informed ants across all runs that remain extant for at least 1 hour after reaching the steady state, $\bar{N_{t_{0}}^{i}}$. The information prevalence is given by the grand mean of this distribution, normalized by the colony size. (**d**) Calculating the probability that the colony loses the information after having acquired it, *P*_*lost*_, which is defined as the proportion of focal runs that were still extant at the mean hitting time $\bar{T}$, but that later decline until no ants are informed (red crosses). For all panels shown here, *β* = 1, *μ* = 0.01.

To investigate how the activity cycles might influence the transmission of information through the colony, simulations inspired by SIS (susceptible-infected-susceptible) models of epidemiological processes were run upon the time-ordered contact networks. Because our focus is the transmission of information rather than disease, for clarity we label the two states ‘uninformed’ and ‘informed’, and refer to the model as the UIU model. This model simulates the two processes of information transmission and information loss as two mutually antagonistic feedback loops (Fig 2a). The model is initiated with one informed ant (in state I), while all others ants are uninformed (in state U). To model information spread, an uninformed ant that makes physical contact with an informed ant can become informed according to a stochastic process controlled by the ‘transmission rate’, *β*. To capture information loss (e.g., through an informed ant forgetting relevant information, or equivalently, through the decay of an information-bearing chemical), informed individuals can spontaneously return to being uninformed according to another stochastic process controlled by the ‘loss rate’, *μ*. The interplay of these competing processes may lead to the population of informed ants either (i) declining to zero, or (ii) growing until reaching a steady state where the rate of individuals becoming informed (U→I transitions) roughly balances the rate of individuals losing the information (I→U). At this point, the information becomes self-sustaining (Fig 3a).

We measured three important components of transmission, namely, (i) the probability that the information reaches a level at which it becomes self-sustaining, *P*_*sustain*_ (Fig 3b), (ii) the prevalence, which is the probability that an ant is in the informed state when the information has become self-sustaining, *P*_*informed*_ (Fig 3c), and (iii) the probability that the colony loses the information after it has become self-sustaining, *P*_*lost*_ (Fig 3d). For formal definitions of these measures, see S1 Text.

### Overview of short- and long-term activity patterns

The colony activity time-series exhibited considerable variation in periodicity both from one hour to the next and also between days and nights (see S1 Text). Therefore, to identify the dominant cycle period, we used wavelet spectral analysis, a time-frequency decomposition that is especially powerful for analyzing nonstationary and quasi-periodic time-series [43, 44]. We use wavelet analysis to calculate a ‘periodogram’ for each time-series. The periodigram depicts the proportion of the overall variance in the time-series that is explained by oscillations with a given period, which is known as the ‘power’. Hence, the periodogram of a time-series with regular oscillations at a given period, will exhibit a peak at that period, and the greater the amplitude of such oscillations, the greater the peak.

These analyses confirmed the presence of short-term collective activity cycles, both in terms of the mean speed of all ants in the nest (‘movement activity’, Fig 1a), and the total number of interactions (‘contact rate’, Fig 1b and 1c). Indeed, the dominant period in the power spectra of the movement activity and contact rate time-series were almost identical (Fig 1e and 1f, S1 Text). Therefore, because we were primarily interested in the properties of the contact network, in what follows we use the periodicity defined by the contact rate time series. The mean and standard error of the cycle period period was *λ* = 19±1.7 minutes, or *γ* = 1/*λ* = 0.0008 cycles per second (Fig 1i), in close agreement with previous estimates [11, 12, 13].

**Fig 4:**
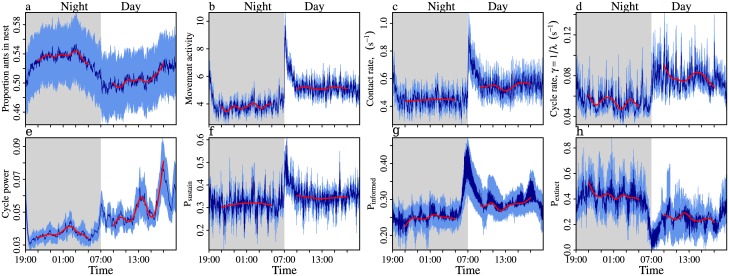
Long-term colony activity patterns. Dark and light blue time series show means and standard errors, aggregated into 1-minute time bins. Each of the 15 recording sessions contributes a single value to each 1-minute bin. Grey background shading—lights off, 20°C. White—lights on, 25°C. Red line—fit from a generalized additive mixed model (GAMM), with the time of the day (time in seconds since the lights were last switched on or off), and time-period (day/night) coded as main effects, and with colony identity (A-F) and replicate number (i,ii) coded as random effects. Due to the disturbance induced by the lights being switched on or off, the GAMM only included times in the range 21:00-05:00 and 09:00-17:00. At night there were more ants in the nest than in the day (**a**). (**b-c**) Ants moved less, and had fewer interactions at night compared to the day. (**d-e**) Oscillations in the interaction rate were slower and weaker at night compared to the day. Note, the wavelet analysis for the movement activity time-series gave identical results as that for the interaction rate. (**f-h**) The ant-to-ant contact network was less efficient at night compared to the day, having a lower probability of becoming self-sutaining, lower prevalence, and higher probability of loss (*β* = 1, *μ* = 0.006).

As well as short-term activity cyles, the colony activity patterns also exhibited diurnal rhythms. Specifically, at night there were more ants inside the nest (Fig 4a, Tukey’s HSD post-hoc comparison of the proportion inside during the night compared to the day; z = 44.6, p<0.00001), and colonies exhibited slower movement (Fig 4b, z = -57.9, p<0.00001) and lower contact rates (Fig 4c, z = -36.6, p<0.00001). Similarly, the nature of the short-term oscillations changed depending on the time of day; at night the activity cycles were both slower (Fig 4d; z = -42.8, p<0.00001), and of lower amplitude (Fig 4e; z = -37.2, p<0.00001) than in the day (see S1 Text). In other words, during the day colonies were more active, oscillating at a higher tempo and with a greater amplitude than at night.

The UIU model simulations showed that the diurnal activity cycles also influenced the contact network transmission properties. At night, information was less likely to become self-sustaining (lower *P*_*sustain*_, Fig 4f, Tukey post-hoc comparison of day versus night; z = -11.2, p<0.00001), whilst information that did become self-sustaining circulated among a smaller proportion of the colony (lower *P*_*informed*_, Fig 4g, z = -28.8, p<0.00001). and had a higher probability of being lost (higher *P*_*lost*_, Fig 4h, z = 33.8, p<0.00001). Hence the transmission properties mirrored the regime of nighttime quiescence and daytime activity; information that is discovered at night should be transmitted slower and less efficiently than information discovered in the day.

### Effect of short-term activity cycles on transmission

**Fig 5:**
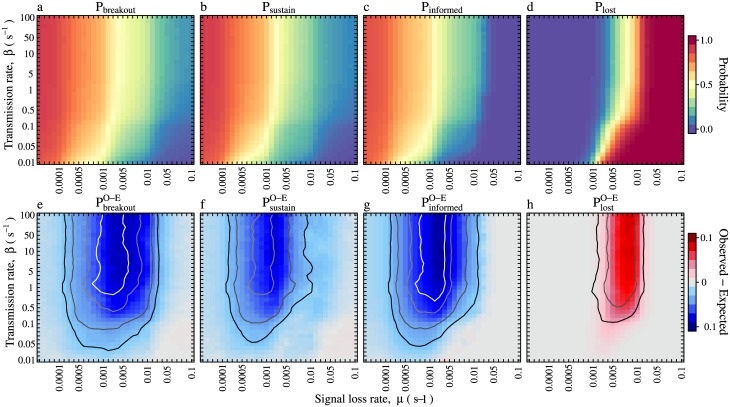
Parameter space explorations show transmission inhibition. **a-d** Parameter space exploration produced by running the UIU model upon the observed contact networks across a range of signal transmission and loss rates. **e-h** Signed difference between transmission over the original time-ordered contact networks (observed) and the synthetic aperiodic networks produced by the null model (expected). (**a,e**) The probability that the information spreads further than the initial seed. (**b,f**) The probability that the information reaches the steady state, and hence becomes self-sustianing. (**c,g**) The size of the steady state population of informed ants, expressed as a proportion of the entire colony. (**d,h**) The probability that the informed population declines to zero. Each {*β*, *μ*} point in the parameter space represents an average across the 15 networks. All contact networks were constructed using the most conservative contact definition, *θ*≥120°. Contours indicate the number of networks for which the observed-expected difference was statistically significant (at p≤0.05). Black contour; ≥3 networks significant. Dark grey; ≥6 significant. Light grey; ≥9 significant. White≥12 significant.

For the parameter-space analysis, in addition to the three transmission metrics described above, we additionally consider the probability that information spreads past the first informed individual (the ‘seed’), which we write, *P*_*breakout*_ (see ‘Quantifying transmission on time-ordered contact networks’ in the Methods). Large numbers of UIU simulations covering a wide range of different transmission- and loss-rate combinations {*β*, *μ*}, confirmed the natural intuition that when information is highly contagious and long-lived (high *β*, low *μ*), it is (i) be more likely to be spread onwards from the initial seed to other ants (high *P*_*breakout*_, Fig 5a), (ii) have a higher probability of becoming self-sustaining (high *P*_*sustain*_, Fig 5b), (iii) reach a wider proportion of the workers when self-sustaining (high *P*_*informed*_, Fig 5c), and (iv) have a lower probability of disappearing (low *P*_*lost*_, Fig 5d). Conversely, when information is non-contagious and short-lived, then it will be less likely to spread further than the seed, less likely to become self-sutaining, reach a smaller proportion of the workers when self-sustaining, and have a higher probability of disappearing.

To test whether STACs influence information flow, for each network and for each of the four transmission components, we calculated the signed difference between the transmission component measured on the periodic observed network and on the aperiodic synthetic network (see ‘Synthetic aperiodic networks’ in the Methods). These comparisons revealed that, contrary to the prediction that information should spread more rapidly within oscillating colonies [11, 13, 26], for all four transmission components information flow was reduced in the observed (periodic) networks compared to the synthetic (aperiodic) networks (Fig 5e–5h).

**Fig 6:**
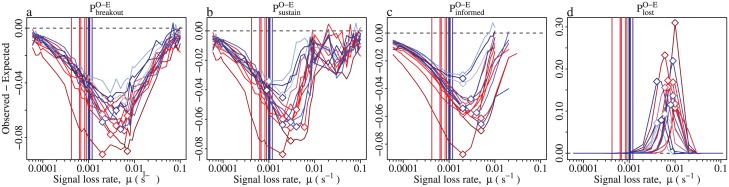
Transmission inhibition is maximal when the loss rate *μ* exceeds the activity cycle rate, *γ*. Each line gives the transmission characteristics for a single three-day contact network as a function of the loss rate *μ*. The transmission rate is held constant at *β* = 1. Coloured lines distinguish among different colonies. Vertical lines indicate the activity cycle rate for each network, *γ* = 1/*λ*. Diamonds indicate the maximum transmission reduction attained by each network. Panels show the difference between observed and expected values of (**a**) the mean probability that the information spreads beyone the initial seed, (**b**) the mean probability that the informed population reaches the steady state, (**c**) the mean size of the steady state population, and (**d**) the mean probability that the informed population declines to zero.

If it is the STACs that are responsible for inhibiting information flow, then we also expect to observe a correlation between the activity cycle amplitude and the magnitude of the inhibition. This would mean that information introduced into a contact network with only a weakly periodic activity cycle, would have roughly the same probability of becoming self-sustaining, reach a similar number of individuals when self-sustaining, and have a similar probability of disappearing, as it does when introduced into an equivalent but completely aperiodic network—as represented by the synthetic networks. Conversely, information introduced into a strongly-periodic contact network, would be impeded, that is, it should have a lower probability of becoming self-sustaining, reach fewer workers when it does become self-sustaining, and be more likely to disappear, than when introduced into the corresponding synthetic network. Therefore, we used mixed-effects regression modelling [45, 46] to test whether the magnitude of the difference in transmission between the original periodic networks and the synthetic aperiodic networks (y-position of the diamonds in Fig 6), depended upon the amplitude of the dominant activity cycle, as given by the wavelet power in the periodograms (the y-position of the diamonds in Fig 1h, for details on the mixed-effects models see S1 Text). Indeed, for all four transmission metrics, this difference displayed a significant positive dependence upon the activity cycle amplitude (*P*_*breakout*_; d.f. = 13, t = -2.4, p = 0.033, *P*_*sustain*_; d.f. = 10, t = -3.1, p = 0.011, *P*_*informed*_; d.f. = 13, t = -2.3, p = 0.038, *P*_*lost*_; d.f. = 9.8, t-3.5, p<0.00001). These statistical associations therefore lend support to the idea that activity cycles inhibit information flow.

The most likely explanation for this association is that the quiescent periods act as barriers to information flow. This mechanism depends upon the relation between the typical lifespan of the information 1/*μ*, and the typical interval between activity peaks *λ*: if information is short-lived relative to the period of the oscillations (*μ* > *γ*), its propagation will be impeded because the time between activity peaks is so long that informed ants entering a quiescent period will likely lose the information before the next activity peak arrives (Fig 2b). Conversely, if the information is long-lived relative to the period of the oscillations (*μ* < *γ*), it will not be impeded because informed ants that enter a quiescent period tend to remain informed until the next activity peak. If this hypothesis is correct, then the transmission reduction should be greater when the lifetime of the information is shorter than the period of the activity cycles (*μ* > *γ*). To test this, for each of the fifteen contact networks, and for each of the four transmission components, we identified the loss rate *μ* at which the reduction was greatest (see diamonds in Fig 6), and then compared this with the activity cycle rate for the same network *γ* = 1/*λ* (vertical lines in Fig 6). As can be seen in Fig 6, in each case the diamonds were always to the right of the vertical lines, confirming the hypothesis that transmission reduction is greatest when the loss rate exceeds the activity cycle rate.

## Discussion

In addition to the prediction of [26] that STACs should facilitate information transmission, several alternative hypotheses have been advanced concerning their function. [12] I suggested that STACs may simply be a side-effect of other processes occurring within the colony, that is, an epiphenomenon with no adaptive value. Others have suggested that they may represent a solution to physiological constraints, such as the build-up of respiratory carbon dioxide within the nest [47]. It has also been argued that STACS should increase ergonomic efficiency because individual workers physically exclude one another in space when performing tasks, hence temporal activity synchronization should provide a more spatially homogeneous distribution of labour [48] However, arguments stemming from physical exclusion have also been used to make the opposite point; that STACs should decrease efficiency because many simultaneously-active workers will cause physical jamming [12].

Irrespective of the ultimate evolutionary origin or the current function of short-term activity cycles, we have shown here that, contrary to the prediction of [26], short-term activity cycles should impede information flow. Further, we provided a mechanism to explain the observed dependence of the magnitude of the effect upon the loss rate *μ*; when the typical lifetime of the information is shorter than the typical duration of the quiescent periods, then information loss during quiescent periods dominates over information spread during active periods.

Our results lead to two different, although not necessarily mutually exclusive, alternatives explanations for the function of STACs. First STACs could represent a mechanism to ensure that outdated or unprofitable information is expunged. For example, when the transmission rate is high relative to the loss rate, then any information spreading within a colony in which the contact rate is constant would take an exceedingly long time to be lost, and so would continue to circulate long after becoming obsolete. Conversely, in a colony with regular activity peaks and dearths, that information would be more rapidly extinguished, allowing the colony to ‘forget’ and allowing room for new information to spread.

Second, given that STACs impede transmission, it is conceivable that they may represent a form of ‘organizational immunity’ [29], that is, a structural (prophylactic) feature of an animal group, that serves to impede the transmission of harmful agents or materials, such as pathogens or poisons [30]. Whilst it has been known for some time that animal groups can attain a degree of organizational immunity by either spatially compartmentalizing a communal nest [49, 50], or by segregating the contact network into different topological subgroups according to age or caste [51, 52], we have shown here that the same outcome might be achieved through a temporal compartmentalization, achieved by constraining activity into regular bursts. However, on balance it seems unlikely that STACs function for such a purpose because the only pathogens that would be inhibited are those that have an infectious period shorter than the time between successive activity peaks (i.e. <20 minutes), which seems unlikely given that the infectious periods of most studied pathogens are orders of magnitude longer [53, 54]. Contrasting the absence of any data on pathogen decay rates in social insects with the abundance of data on rapidly-decaying pheromones in ants and other social insects [19, 55, 56], it is prudent to favour the alternative hypothesis—that short-term activity cycles mainly have a modulatory role in communication.

Finally, although our use of epidemiology-derived models of transmission over networks has provided new insights into the function of a self-organized collective behaviour, there is still much that remains to be drawn from epidemiology. Whilst studies of disease transmission have shown that within-host competitive interactions between distinct pathogen strains determine strain transmission success and evolution of new strains [57], the potential for competition or interference between distinct information streams also exists within societies of humans and other animals, as when true information is contradicted by false or misleading information, or when a group must form a consensus for one among several different options. As more elaborate SIS models for simulating between-strain interactions now exist [33], we look forward to further insights that they will bring to animal social behaviour.

## Methods

### Experiments

Nine queenright *Leptothorax acervorum* colonies were collected from Anzeindaz, Switzerland, in September 2013, and housed in nests with internal dimensions 63x42x2mm. Each colony consisted of a population of sterile workers (mean and standard error; 77±18), a single sterile queen, and a population of brood (116±59). Each ant in each colony was individually labelled with a 0.7x0.7mm ARtag barcode [58], printed on synthetic polymer paper (Orell Füssli Security Printing Ltd, Zurich, Fig 1a).

Throughout the experimental period, the humidity was set to 70%RH. During the day (07:00-19:00) the nest and the foraging arena were illuminated with visible light and the temperature was set to 25°C, whilst at night (19:00-07:00) there was no visible light, and the temperature was reduced to 20°C. For further details on the tagging procedure, the nest and arena design, see S1 Text.

After the arena was placed in the tracking box, 24-hours were allowed to elapse before the tracking was initiated. Thereafter, the identity, position and orientation of each tag within the nest was continuously recorded for 3 days. Colonies underwent two 3-day (72 hour) recording sessions (‘replicates’), with a two-week interval between the end of the first replicate, and the start of the second. However, for colonies 12, 14 & 18, the second replicate failed due to technical problems. Although all recording sessions were used for the formal statistical tests, re-running the same tests on a reduced dataset that excluded the unpaired runs gave similar results.

### Detecting contacts between ants

In this section, we outline how ant-to-ant contacts are detected. Pairwise ant-to-ant contacts were inferred using the method of [31], which models each ant as trapezoid, with an ‘interaction point’ at the front end (Fig 1a). A contact between two ants is initiated when the interaction point of (at least) one of them enters into the trapezoid of the other, and when the orientation difference between their bodies exceeds a threshold angle, *θ*. The contact is terminated at the moment that both ants’ interaction points are outside one another’s trapezoids. As there is evidence to suggest that interactions in which at least one individual makes a head-on contact with the body of another ant, such as trophallaxis, or antennation, do serve for the purpose of information transmission [21, 22, 23, 24], all of the information transmission simulations were run upon networks in which individuals are connected by edges that represent head-to-body interactions, *θ*≥120°. For a demonstration that these automatically-detected contacts did correspond to contacts detected by a human observer, see S1 Text.

### Measures of activity

In order to obtain a measure of activity at the colony level, previous studies have used an effective but basic proxy measure: the pixel difference between successive images [11, 12, 13, 59]. As the automated tracking system provides access to both the trajectories of individual ants, and the ant-to-ant contact sequence, we instead define two measures of colony activity that are based upon these individual-level measures. First, the ‘movement activity’ is the mean of the instantaneous speeds of all ants detected in any given frame, where the instantaneous speed for an single ant is simply the distance it travelled since the last frame (Fig 1a, S1 Video). Second, the ‘contact rate’ is the sum of all ant-to-ant contacts starting or stopping in any given frame (Fig 1b and 1c). Although these time-series are often correlated, there may be occasions when that is not the case, so they were considered independently.

### A simulation model for information transmission

In our model of information transmission, at any given time each ant may be either uninformed (U) or informed (I). We term this the UIU model. Transmission is modelled as a ‘snowball’ process whereby information is transferred (with a constant probability per unit time and maximally once per contact) from one ant in state I to another in state U during physical contact, resulting in two informed individuals where before there was only one, U+I → 2I (Fig 2). For each simulation, all ants are initially labelled U. To represent the arrival of new information, a single ‘seed’ ant is randomly selected, and relabelled I. During contact between an uninformed and an informed ant, the former becomes informed (U+I → 2I) according to a Poisson process with a rate *β* per unit time [60]. We assume that the informed ant can transmit maximally once per contact.

To date, most models of information transmission on social insect contact networks have made the strong assumption that every contact between informed and uninformed individuals always results in transmission [31, 61]. However, physical contacts represent a rather noisy communication channel [25], hence these deterministic models overestimate transmission efficiency. Our use of a stochastic (Poisson) process avoids these assumptions; when *β* is low, contacts between U & I ants rarely result in transmission, whereas when *β* is high, such contacts always result in transmission and the deterministic transmission models are recovered.

Finally, our model goes beyond simple snowball models of information transmission in that we included an additional negative feedback to capture attenuation of the spreading agent. For example, an information carrier (such as an ant) may forget what it has learnt [62, 63], or the information-bearing chemical may decay by itself [19, 64]. Information loss is modelled as another Poisson process with rate *μ*, but unlike transmission (which only occurs during contacts), an informed ant may perform the I→U transition at any time.

The UIU model was implemented in Python, and is freely available [65].

### Quantifying transmission on time-ordered contact networks

To our knowledge, all studies of the transmission properties of animal contact networks have measured the spread of some agent introduced at a single point in time—typically one of the first recorded contacts in the time-ordered network [31, 51, 61]. Whilst this is a reasonable approach when the contact rate and the number of interacting individuals are stationary over time, it is inappropriate when they are non-stationary, because then the measured transmission properties will be entirely contingent upon the particularities of the chosen starting time at which the UIU simulations are initiated and the transmission properties measured. Because the *L. acervorum* contact networks are manifestly non-stationary, they cannot be characterised merely by measuring the transmission from a single time point; instead, for each network we repeatedly sample the transmission properties at a sequence of regularly-spaced starting times, *t*_0_ (see S1 Text for further details).

When using SIS-like epidemiological models to characterise the transmission properties of a particular network, it is important to identify the point at which the information has become self-sustaining, that is, when it has reached a roughly the steady state [33]. For static networks the identification of the steady state is trivial; because the network topology is static, after an early growth period, the mean number of infected (or informed) individuals will converge to a fixed point. However, in a time-ordered network the topology changes over time, hence the mean number of informed individuals also changes over time. We therefore define a time-explicit steady state by identifying the mean time taken for the focal population of informed individuals carrying information introduced at time *t*_0_ to converge to that of a reference population carrying information introduced previously at *t*_0_−*τ*, where *τ* is of sufficient length that the extant reference runs have reached the steady state by the time that the focal runs are initiated at *t*_0_ (Fig 3).

We measure the following four quantities, doing so at each starting time *t*_0_, and with reference to our time-explicit steady state, as just defined. First, *P*_*breakout*_ is the proportion of UIU simulations initiated at a given starting time, *t*_0_, that spread beyond the single initial ‘seed’ individual. Second, *P*_*sustain*_ is the proportion of UIU simulations initiated at *t*_0_ that reach the steady-state (Fig 3). Note, *P*_*sustain*_ includes all simulations that reach the steady state, irrespective of whether they later decline until there are no informed ants. Third, the prevalence is the mean proportion of the workers that are in state I among those simulations that reach the steady state, *P*_*informed*_. The prevalence provides a useful measure of the network transmission efficiency. Last, we define the probability that the information is lost, *P*_*lost*_. The purpose of this measure is to quantify the ability of the entire colony to preserve an information-bearing signal. Preserving a signal means that once the colony has acquired it, the colony does not lose it. However, because the first infected seed ant will often lose the information without ever passing it on to other ants, the principal determinant of the time until the signal disappears will often be the signal loss rate *μ*, and not colony-level features of the contact network. Therefore, we define *P*_*lost*_ by the proportion of simulations that disappear *after having reached the mean hitting time* $\bar{T}$ (Fig 3b). By stipulating that simulations must survive until the point at which most extant simulations have become self-sustaining, $\bar{T}$, those in which the seed never passes on the information are excluded. This ensures that *P*_*lost*_ is defined by information loss among the remaining simulations, in which information was in general circulation within the colony. Hence *P*_*lost*_ quantifies the ability of the entire colony to support the long-term persistence of a signal.

Although these measures may be correlated they are not redundant, for example *P*_*breakout*_ is not analogous to 1-*P*_*lost*_ because, whereas *P*_*lost*_ reflects the ability of the overall contact network to preserve information once it has ‘escaped’ from seed, *P*_*breakout*_ will very often be defined primarily by runs in which the information never spread further than the seed. Hence, *P*_*lost*_ reflects the overall transmission properties of the contact network, whereas does not. Similarly, *P*_*sustain*_ and *P*_*informed*_ differ because an information-bearing signal may have a low probability of becoming self-sustaining, but nevertheless still achieve a high prevalence on those rare occasions when it does so. The same is true of *P*_*informed*_ and *P*_*lost*_, because a signal that achieves only a low prevalence may nevertheless still persist in the population for long periods without declining until no ants are informed. For formal definitions of these measures, see S1 Text.

### Synthetic aperiodic networks

In order to asses whether a particular statistical feature of the observed contact network—in this case, the short-term activity cycling—influences its overall transmission properties, it is necessary to compare the observed transmission with that expected in the absence of the feature of interest, or in other words a null model is required [42]. To that end, we developed a permutation procedure inspired by the time-shifting method of [66], which generates synthetic time-ordered contact networks that lack periodicity, but which are otherwise identical to the original. Briefly, the procedure involves an independent temporal shifting of the sequence of contacts on each edge, where an edge is defined as a unique pair of ants that interacted at least once during the 3-day period (see S1 Text). Because the time-shifting is limited to a maximum forwards or backwards shift of ±*λ*/2, and because a different shift is applied to each edge, the STACs are destroyed, whilst long-term cycles—such as the diurnal cycle—are preserved (see S1 Text for further details).

## Supporting information

S1 Text This text provides additional information on the experiments, and the analyses described in the main text. (PDF)

S1 Video This video shows the characteristic activity cycle behaviour for 2.5 hour period from a single colony. (MP4)
