# Supplementary material for: Short-term activity cycles impede information transmission in ant colonies
Source: PLoS Comput Biol. 2017 May 10;13(5):e1005527. doi: 10.1371/journal.pcbi.1005527 (PMC5443549; doi:10.1371/journal.pcbi.1005527)
Supplement: S1 Text — (PDF) [file pcbi.1005527.s001.pdf]

Supplementary Information: Short-term activity cycles impede information transmission in ant colonies

Thomas O. Richardson, Jonas I. Liechti, Nathalie Stroeymeyt, Sebastian Bonhoeffer, Laurent Keller

1 Experimental details

The tagging procedure involved two steps. The subject ant was first anaesthetised by brief (∼5-10 sec) immersion in a CO_2 bath, and then removed and placed in a crevice cut into a soft sponge, allowing access to its thorax. The sponge was then placed back into the CO_2 bath, and the ARtag immediately attached to the dorsal thorax surface (Fig. S1) using a small drop of adhesive (Pattex Power Easy Gel). Each ant typically experienced less than 30 seconds in the CO_2 bath, and other than a temporary increase in grooming behaviour, the tags induced no apparent bias in behaviour, movement or interactions.

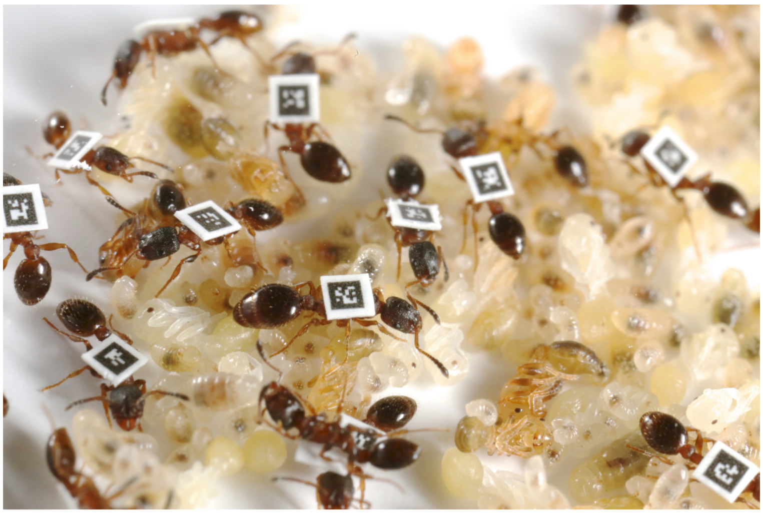

Figure S1: *Leptothorax acervorum* workers and brood. ARtag barcode markers are attached to the adult workers. Tags are printed onto synthetic paper and fixed to the thorax with glue. Photo courtesy of James Waters.

Each colony was housed in a nest composed of a cardboard gasket wall (63x42x2mm internal dimensions), sandwiched between a transparent glass slide at the bottom and an infra-red transmitting filter glass slide (R-64, Hoya, USA) at the top. The infra-red transmitting filter blocked most visible light and only allowed wavelengths longer than 640nm to pass through. To prevent tagged ants climbing onto and walking upon the nest roof, the nest was surrounded by a 25mm enclosure, whose outer surface was coated in Fluon. The nest and enclosure were placed in a 130x190mm arena, which the ants could access via a tunnel in the enclosure.

Twelve hours prior to the start of the recording, the entire arena assembly was placed within a foam tracking box in which the temperature, humidity, and visible light regimes were carefully controlled. During the day (07:00-19:00) the tracking box was illuminated with visible light and the temperature was set to 25^oC, whilst at night (19:00-07:00) there was no visible light, and the box was cooled to 20^oC. Although at night there was no visible illumination, tracking could still proceed via the infra-red illumination, which the camera could detect but which ants could not (Briscoe and Chittka, 2001). Despite the visible illumination during the day, the infra-red filter slides on the nest roof and floor allowed only infra-red light into the nest chamber, hence the nest appeared dark to the ants. The foam box also contained high-resolution (4560x3048 pixels) infra-red sensitive video camera, which captured two frames every second. This camera was connected to an array of infra-red LEDs, which flashed twice every second to coincide with the camera exposure, allowing the detection of tags even when the visible lights were switched off (Mersch et al., 2013). At all times, protein (*Drosophila*), sugar water, and drinking water were provided *ad libitum*. For each replicate, the colony demography (the number of workers and brood) was assessed using a daily census; the 3-day means are reported in Table S1.

2 Contact validation

In this section we perfom two comparisons, both of which quantified the agreement between the contacts detected by the automatic ARtag tracking system, and manually-detected contacts, as identified by a human observer. The first comparison involved the manual identification of 50 head-to-head contacts occurring during the 2.5 hours of the recording session shown in Fig. 1e-g in the main paper. For each manually-identified contact, the identities of both ant participants were noted, as well as the times that the contact began and ended. These manual contacts were compared with the automatically detected head-to-head contact sequence from the same period. Of the 50 manual contacts, 47 were also present in the automatic contact sequence, which corresponds to a 94% match.

Conversely, in the second comparison we randomly selected 50 automatically-detected contacts from the same 2.5 hour period. Using the start ant stop time of these 50 contacts, we then visually checked the corresponding video recording to see whether they were visible to the naked eye. Of the 50 automatic contacts, 45 were also seen in the videos, which corresponds to a 90% match.

3 Identification of the dominant period using wavelet decomposition

Previous studies of the oscillatory activity patterns in *Leptothorax* colonies have used Fourier spectral analysis to identify the dominant period in the time-series of colony activity (Cole, 1991; Cole and Cheshire, 1996; Cole and Hoeg, 1996; Boi et al., 1999). However, because Fourier spectral analysis is a frequency decomposition, rather than a time-frequency decomposition, it is only applicable to stationary time-series, that is, those in which both the dominant frequencies do not change over time. Indeed, Fourier spectral analysis may produce misleading results when applied to non-stationary time-series.

As the two indicators of colony activity (movement and contact rate) displayed consistent differences both within and between days and nights (Fig. 4 in the main paper), we used an alternative technique – wavelet spectral analysis. Wavelet analysis is robust to time-series that are non-stationarity because, unlike Fourier spectral decomposition, at each time-point within the time-series it provides a breakdown of the dominant periods, which is referred to as the 'power spectrum' (Percival and Walden, 2006; Cazelles et al., 2007; Roesch and Schmidbauer, 2014). Thus, for each half-second throughout the three-day observation period, the wavelet time-frequency decomposition provides, (i) the dominant cycle length, λ, and (ii) the corresponding amplitude (wavelet 'power') for the dominant period.

These analyses confirmed that the dominant period length and the power of the oscillations varied both within and between days and nights (Fig. S2a), with days exhibiting shorter and more powerful oscillations than nights (Fig. S2b-d, Table S1). The overall wavelet power spectra for the movement activity and contact rate time series for the 15 recording sessions are shown in Fig. S3.

Table S1: **Summary of demographic, topological and temporal characteristics for each 3-day replicate**. The column labelled 'realized edges' gives the proportion of unique ant pairs that interacted at least once. The column labelled 'N contacts/edge' gives the mean number of contacts on each (realized) edge. The last two columns give the dominant period (λ, in seconds), and the corresponding wavelet power (amplitude), of the 3-day contact rate time-series. Values in parenthesis indicate the same for the day and night.

Colony	Replicate	N workers	N brood	N contacts	Realized edges	$\langle$N contacts/edge$\rangle$	λ (day,night)	Power (day,night)
2	2	57	102	148 276	0.99	94	1594 (1040,1604)	0.014 (0.017,0.014)
2	1	75	103	105 430	0.83	46	939 (904,1204)	0.014 (0.017,0.013)
5	2	82	142	121 158	0.84	44	988 (864,1130)	0.013 (0.016,0.014)
5	1	97	192	315 919	0.92	73	832 (795,1087)	0.045 (0.068,0.049)
6	2	80	118	276 147	0.99	88	1020 (994,3016)	0.019 (0.029,0.017)
6	1	81	138	338 802	1	105	945 (939,1342)	0.05 (0.092,0.023)
8	1	28	23	55 626	0.97	151	1014 (957,1144)	0.019 (0.022,0.02)
8	2	62	73	77 468	0.89	46	969 (904,1087)	0.012 (0.017,0.009)
10	1	64	58	279 612	0.98	142	1130 (1101,1235)	0.049 (0.065,0.037)
10	2	56	33	176 676	1	115	2382 (2382,2367)	0.025 (0.023,0.028)
12	1	100	256	311 992	0.95	67	842 (832,875)	0.014 (0.019,0.01)
13	1	84	156	365 712	0.99	106	982 (945,1333)	0.026 (0.04,0.024)
13	2	81	139	199 287	0.94	66	1564 (1094,1594)	0.016 (0.02,0.018)
14	1	54	94	142 057	0.98	101	1477 (933,1584)	0.014 (0.013,0.019)
18	1	66	112	226 228	1	106	1220 (1220,1220)	0.019 (0.025,0.013)

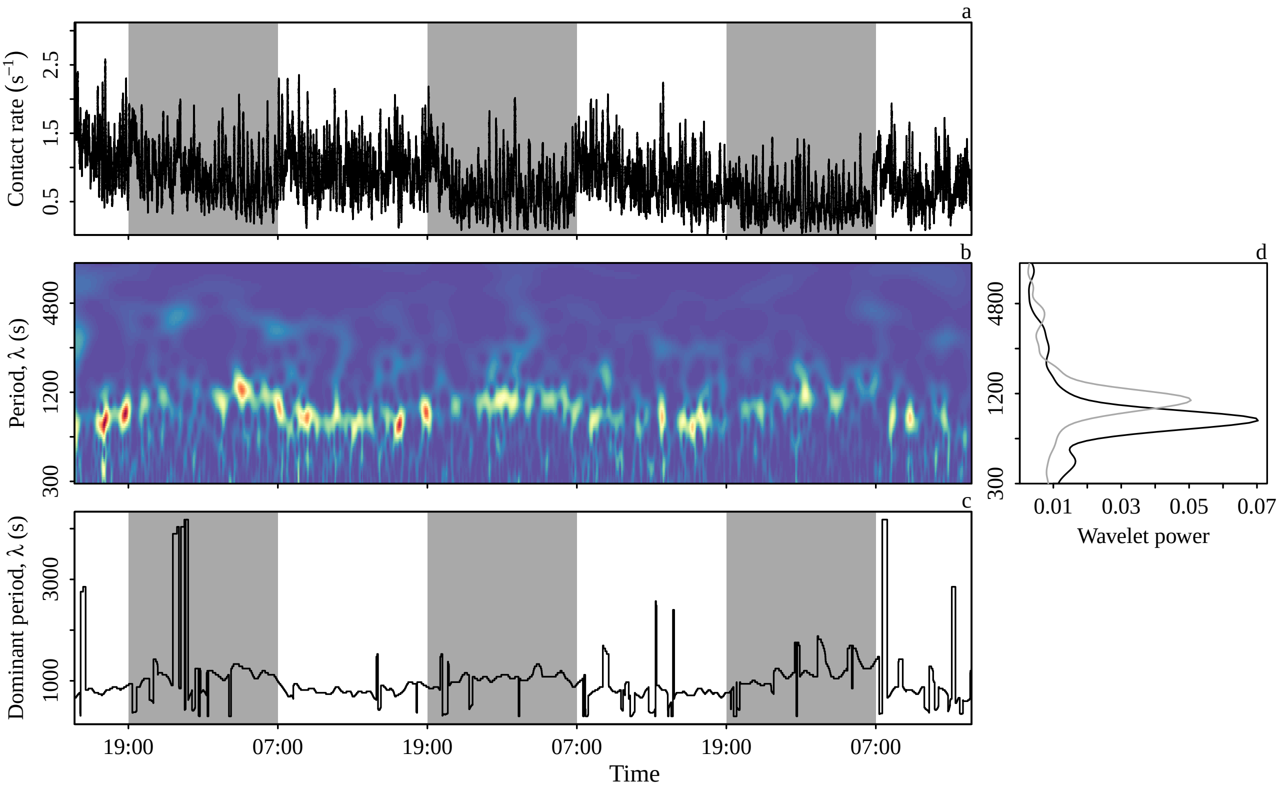

Figure S2: **Identifying the dominant period in the contact rate time-series.** (**a**) The 3-day contact rate time-series for one replicate (colony 5, replicate 1). Nights are indicated by the grey shading. (**b**) Wavelet time-frequency decomposition of the contact-rate time-series. Colours encode the wavelet power, that is, the proportion of the overall variation in the time-series explained by a particular period, λ. Periods that are dominant in the time-series appear in red. (**c**) The dominant period over time, where the dominant period is defined as the period with the greatest power at each time-step. (**d**) The overall wavelet power spectrum is obtained by aggregating the temporal wavelet power spectrum across all time-steps. Black line - days, grey line - nights.

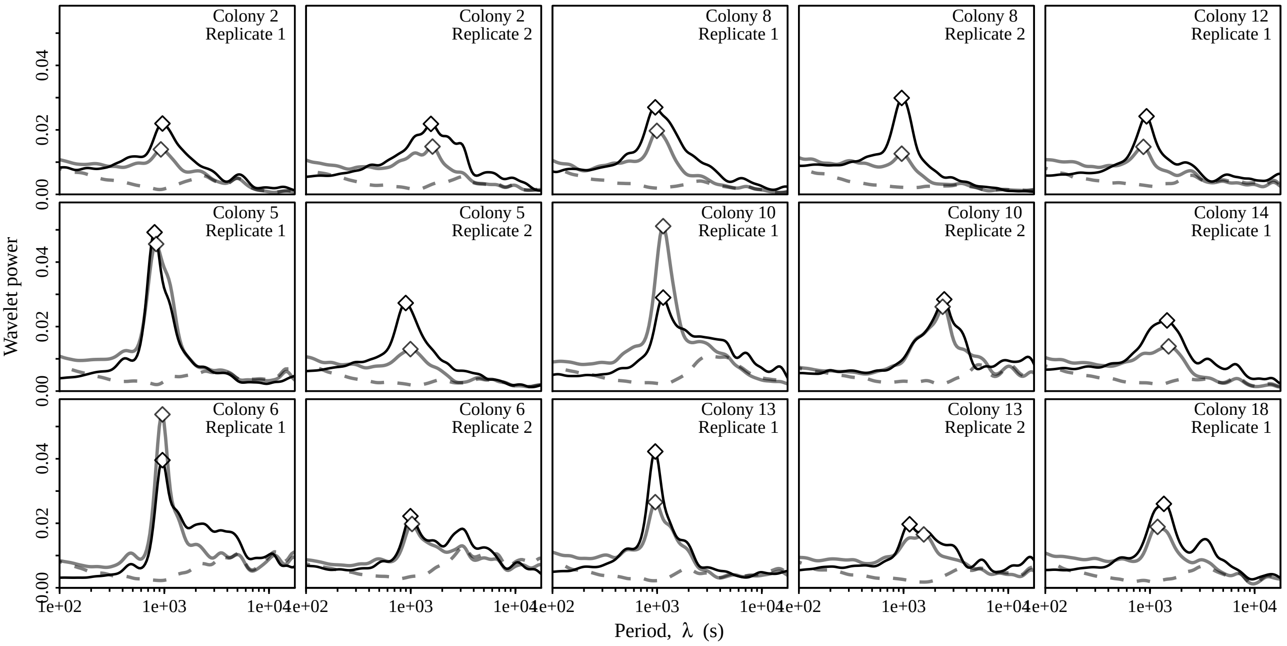

Figure S3: **Wavelet power analysis confirms the presence of colony activity cycles, with a ∼20-minute period.** Each panel depicts the wavelet power spectrum (periodogram) for a single 3-day replicate. Solid black line: Periodogram for the movement activity time-series. Solid grey line: Periodogram for the observed contact rate time-series. Dashed grey line: Periodogram for contact rate in the synthetic networks produced by the period-shift null model. Diamonds indicate the period with the greatest intensity (wavelet power) within the time-series.

4 Synthetic networks without short-term activcity cycles

The method used to produce the synthetic null model networks that lack short-term activity cycles is a permutation procedure that requires two inputs, (i) the time taken between two activity peaks λ, and (ii) the observed temporal contact network. For each edge (i.e. each ant pair), a time is randomly sampled from a uniform distribution in the range $y \in [-\lambda/2, \lambda/2]$, and all the contacts on that edge are shifted forwards (when $y > 0$) or backwards ($y < 0$) by that amount (Fig. S4c,f). Because y is independently sampled for each edge, the activity on any given edge becomes decoupled from that on other edges, hence the resulting temporal contact network lacks short-term periodicity (Fig. S4b,e). By constraining y to a relatively short time ($\lambda/2$), temporal fluctuations occurring on longer timescales such as the diurnal variation in the contact rate, are conserved (Fig. S4a,d). The absence of short-term activity cycles from the synthetic contact networks was confirmed by the wavelet power spectra of the contact rate time series (dashed lines in Fig. S3).

Futhermore, because the contacts on each edge are subjected to a temporal shift rather than randomly reallocated among individuals as in other null models based on edge 'rewiring' (Isella et al., 2011; Holme and Saramäki, 2012), these synthetic networks conserve several topological properties that are key in determining overall transmission properties, such as the degree distribution, degree correlations, and the overall community structure (Newman, 2002). Additionally, the period shifting procedure conserves the distribution of the waiting times between contacts on each edge, which can also influence overall transmission properties (Karsai et al., 2011; Perotti et al., 2014). For these reasons, any differences between the original and synthetic networks, may be attributed to the short-term activity cycles.

5 Information transmission model implementation

The UIU model dynamics are simulated using a Gillespie next reaction method (Gibson and Bruck, 2000) leading to a continuous process. An ant that undergoes the transition U→I at time t^*, is assigned an informed duration dt, drawn from an exponential distribution with recovery rate parameter μ, and a relabeling event for this ant at time $t^* + dt$ is written into a priority queue. Then, for all interactions of this ant that have an end time t_{end} bigger than t^* and a start time t_{start} smaller than $t^* + dt$, a time to transmission dt_{trans} is drawn from an exponential distribution with information transmission rate parameter β. If the time to transmission is smaller than the duration of the interaction for which the informed ant stayed informed, that is, if $dt_{trans} < min(t_{end}, t^* + dt)$, a relabelling event for the interaction partner is written into the priority queue

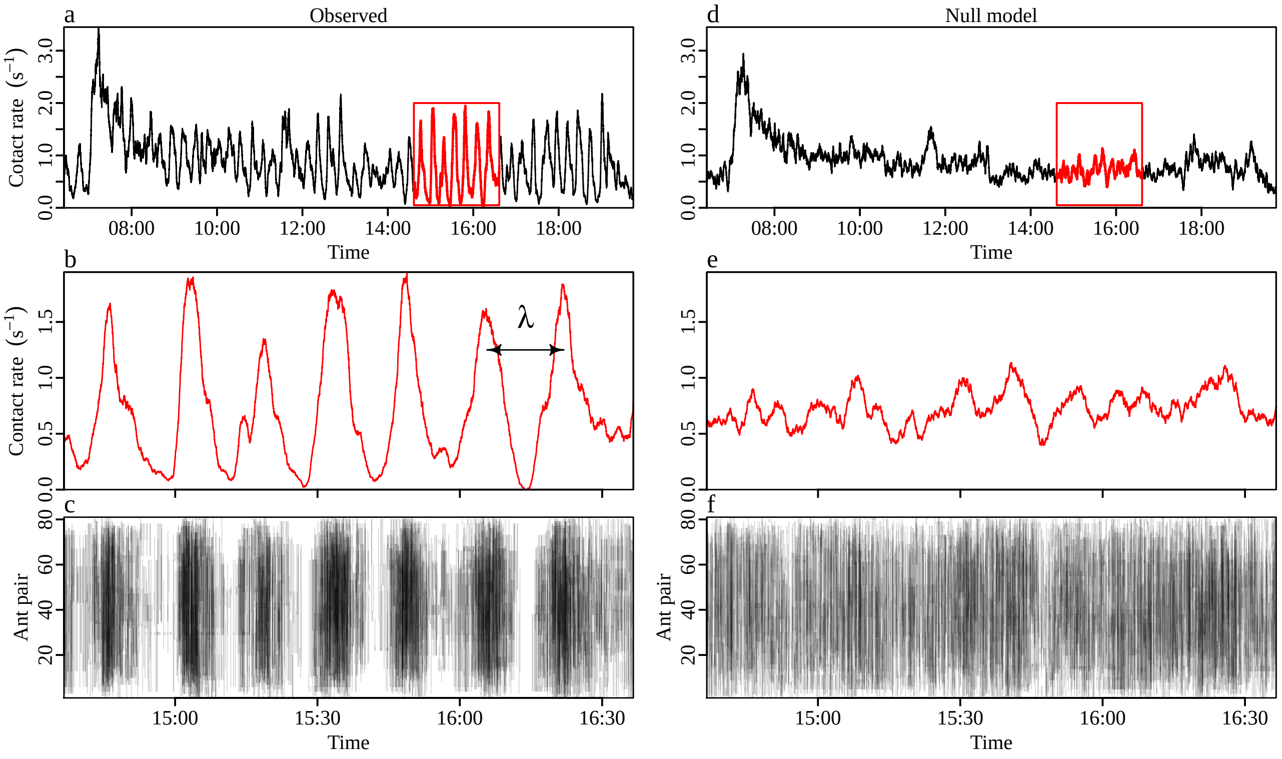

Figure S4: **Period-shifting procedure for generating aperiodic contact networks.** (**a**) Time-series of the observed total number of interactions occurring every half-second over one day (colony 6, replicate 1). (**b**) Blowup of a particularly cyclical two-hour period, indicated by the red highlights in panel **a**. Contacts are clustered in time, occurring regularly with a period of λ=16.2 minutes. (**c**) The temporal contact network for the same period. Black vertical lines represent the start of a physical contact between two ants (contact duration omitted for clarity). Activity peaks and quiescent periods are indicated by the alternating pattern of contact bursts and dearths. (**d-f**) In the period-shift null model, the interactions occurring within each ant pair are randomly shifted forwards or backwards in time by sampling a random shift y from a uniform distribution in the range $y \in [-\lambda/2, \lambda/2]$. (**d**) This period-shifting preserves temporal features occurring on timescales greater than λ, such as the morning increase in the interaction rate. (**e**) Period-shifting removes the short-term activity cycles, as the interactions occurring between different ant pairs (**f**) are no longer synchronized.

with a time of occurrence $t_{start} + dt_{trans}$. At this point the next label change is drawn from the priority queue. If the relabeling is from informed to uninformed (I→U), the status of the partner ant being considered is updated and no new events are written into the priority queue. If the relabeling is from uninformed to informed (U→I) two cases are distinguished: i) If the ant in question is already informed I, the event is simply ignored. ii) If it is uninformed U, the process described above is carried out again. A simulation ends if either the observation period ends or the priority queue is empty, in which case the information was lost.

6 Formal definitions of $P_{breakout}$, $P_{sustain}$, $P_{informed}$, and P_{lost}

When UIU models are applied to a static contact network, the agent typically exhibits an initial growth phase and then settles into a stable equilibrium – a so-called 'steady state' with a fixed mean and variance. However, unlike in a static contact network, in a time-ordered network both the topology and interaction rate can fluctuate over time. If these fluctuations are non-stationary—as here—then the 'steady state' is not steady at all; in that case, we may instead define a dynamic 'quasi-steady-state' (dQSS). A single UIU run initiated at time t_0, is defined as having reached this dQSS when the number of informed individuals at a later time t, that is $N_{t_0}(t)$, equals or exceeds the mean number of informed individuals at time t for an ensemble of runs that were initiated at an earlier time $t_0 - \tau$, that is when $N_\tau(t) \geq \langle N_{t_0-\tau}(t) \rangle$, where the angular brackets indicate an average over the ensemble initiated at $t_0 - \tau$. As long as time interval between the two starting times, τ, is sufficiently long, we can be sure that the surviving runs within the ensemble $\langle N_{t_0-\tau}(t) \rangle$, have already reached the dQSS by time t.

In what follows we provide formal definitions of the dynamic quasi steady state, dQSS, and the four probabilities used in the analysis, $P_{breakout}$, $P_{sustain}$, P_{lost}, and $P_{informed}$. Although these measures have previously been applied to time-aggregated (static) networks (Leventhal et al., 2015), they have yet to be defined for time-

ordered networks. Hence, we now formally define these four measures within an explicitly temporal context.

As the contacts in a time-ordered contact network are spread over time, so the transmission properties vary according to the time at which transmission is measured. Therefore, all four quantities will be a function of the time at which we start the simulations and a later time at which we assess those quantities, e.g. $P_{sustain} \rightarrow P_{sustain}(t_0, t)$ where t_0 is the time at which simulations started and $t \geq t_0$ the time at which we assess the probability that the information becomes self-sustaining. In order to simplify notations we provide the formal definitions for a fixed starting time, t_0, of the simulations. It follows that e.g. the number of informed individuals at a time $t \geq t_0$ is written as $N(t)$ and the number of informed in a simulation starting at an earlier time, $t_0 - \tau$, is given by $N_{-\tau}(t)$.

Let $S \in \mathbb{N}$ be the number of simulations initiated at time t_0. Let I_0^i be the set of the initially informed individuals in the ith simulation and let $I^i(t)$ be the set of informed individuals at time t. We denote $N^i(t)$ as the number of informed individuals at time $t \geq t_0$ for the ith simulation initiated at time t_0, where $i \in [\![1, S]\!]$. First we define a function d labeling a run i if the information ever reached individuals that were not in the initial set of informed individuals:

$$\begin{aligned} d :&[\![1, S]\!] \longrightarrow \{0, 1\} \\ &i \longmapsto \begin{cases} 1, & \text{if } \exists t' \geq t_0, I^i(t') \neq I_0^i \text{ and } I^i(t') \neq \emptyset \\ 0, & \text{otherwise} \end{cases} \end{aligned}$$

The probability of breakout, $P_{breakout}$, can now be defined as the ensemble of runs in which the information is still present and the ensemble of informed individuals is different from the ensemble of initially informed individuals divided by the total number of simulations:

$$P_{breakout} = \frac{|\{i \in [\![1, S]\!] | d(i) = 1\}|}{S}$$

Now we define a function f that, at a time t, indicates whether there are no more informed ants in run i as:

$$\begin{aligned} f_i :&\mathbb{R}^+ \longrightarrow \{0, 1\} \\ &t \longmapsto \begin{cases} 1, & \text{if } N^i(t) \neq 0 \\ 0, & \text{otherwise} \end{cases} \end{aligned}$$

The ensemble of runs in which the information is still present at time t can then be defined as:

$$U(t) = \{i \in [\![1, S]\!] | f_i(t) = 1\}$$

To define the probability that the information becomes self-sustaining, $P_{sustain}$ we start with defining the dynamic quasi steady state, dQSS, at time t as:

$$dQSS(t) = \overline{\{N_{-\tau}^i(t)\}_{i \in U_{-\tau}(t)}},$$

which is the mean number of informed for all the runs starting at time $t_0 - \tau$ in which the information was not lost up to the time t. We then further define the function b for each run i as:

$$\begin{aligned} b_i :&\mathbb{R}^+ \longrightarrow \{0, 1\} \\ &t \longmapsto \begin{cases} 1, & \text{if } \exists t', t \geq t' \geq t_0, N^i(t') \geq dQSS(t') \\ 0, & \text{otherwise} \end{cases} \end{aligned}$$

so $b_i(t)$ labels simulation i as having reached (label 1) or not (label 0) the dynamic quasi steady state at some time up to the time t. We can then define the ensemble of simulations that ever reach the quasi steady state as:

$$W = \{i \in [\![1, S]\!] | \exists t \geq t_0, b_i(t) = 1\}$$

The probability that the information becomes self-sustaining is then the fraction of the starting runs that ever reach the dQSS:

$$P_{sustain} = \frac{|W|}{S}$$

To define the information prevalence, $P_{informed}$, we start with the hitting time, T_i, of run $i \in W$ as the minimal time it took the run to first hit the quasi steady state:

$$T_i = min(\{t \in \mathbb{N}, N^i(t) \geq dQSS(t)\})$$

And thus the mean hitting time for simulations starting at time t_0 as: $\overline{T} = mean(\{T_i\}_{i\in W})$.

The prevalence for simulation, $i \in W$ is given as the mean number of informed over a certain amount of time, Δt after the hitting time:

$$P^i_{informed} = \overline{\{N^i(t)\}_{t\in[T_i,T_i+\Delta t]}}$$

Finally, the prevalence at time t_0 is then given by the mean prevalence of all simulations that reached the dynamic quasi steady state:

$$P_{informed} = mean(\{P^i_{informed}\}_{i\in W}).$$

At last, we define g as a function labeling a run i if the information will be lost (label 1) or not (label 0) at some time after t as:

$$\begin{aligned} g_i :&\mathbb{R}^+ \longrightarrow \{0,1\} \\ t \longmapsto &\begin{cases} 1, & \text{if } \exists t' \geq t, N^i(t') = 0 \\ 0, & \text{otherwise} \end{cases} \end{aligned}$$

and the ensemble of runs that lose the information at some time point after time t as:

$$V(t) = \{i \in U(t) | g_i(t) = 1\}$$

The probability that a run declines until no ants are informed at time t is then defined by the number of runs that preserve the information up to time t but subsequently lost it:

$$P_{lost}(t) = \frac{|V(t)|}{|U(t)|}$$

Note that this definition only imposes $t > t_0$ as a condition on the time point t at which P_{lost} is measured. So in principle it would be possible to choose $t \approx t_0$ or $t >> \overline{T}$. However, if we want to assess the likelihood of losing information introduced at time t_0, choosing t close to t_0 is not ideal, as the closer to t_0 we get, the smaller the fraction of the population the information could possibly have reached during the time $[t_0, t]$. Therefore, to get a sensible estimate for the probability of information loss at the population level, it is desirable to choose a large t. On the other hand, if we choose $t >> \overline{T}$ the measure of information loss becomes increasingly disconnected from the time when the information was introduced t_0, because the probability of information loss is determined by the dynamics during $[t_0, t]$. Since the occurrence of any quiescent periods within $[t_0, t]$ will contribute to P_{lost}, the bigger $t - t_0$ the bigger the ensemble of starting points for which this period will have an impact on P_{lost}, and the more we lose the temporal dimension of the P_{lost}. As a compromise between a highly time-localized measure, and a temporally-extended measure that loses the temporal aspect of the measure, we chose the mean hitting time as the time point at which to assess the probability of extinction; it is the soonest time point at which we can expect the information to have reached abundance levels relevant to the population.

7 Quantifying transmission

In order to ensure that the estimated transmission characteristics of the contact networks are independent of the particular time(s) at which the UIU simulations are initiated, all the transmission analyses presented in the main paper were based upon a sequence of starting times, t_0, spread evenly across the 3-day observation period, with successive starting times sparated by Δt=240 seconds.

As two of our transmisison metrics – the proportion of runs that become self-sustaining ($P_{sustain}$), and the proportion that decline until no ants are informed (P_{lost}) – are probabilistic measures, derived from the outcomes of a population of UIU runs, it is necessary to initiate multiple runs at each starting time t_0. Therefore an ensemble of 100 UIU simulations was initiated at each t_0. This repeated measurement of the contact network transmision properties at multiple regularly-spaced starting times t_0 produces three time-series for each $\{\beta, \mu\}$ combination – one for each of the three measures of transmission. Across-network averages of these time-series are given in Fig. 4 in the main paper. However, in order to examine how different combinations of the transmission rate β and the loss rate μ influence transmission, it is necessary to condense the time-series for each $\{\beta, \mu\}$ combination into a single estimate. Therefore to produce Fig. 5-6 in the main paper, we took the mean of each time-series, thus arriving at a single estimate for each of the four metrics, $P_{breakout}, P_{sustain}, P_{informed}$ and P_{lost}, that evenly weights transmission throughout the entire contact sequence. Note that to avoid presenting 15 transmission parameter spaces for each of the four variables, each $\{\beta, \mu\}$ combination in Fig. 4 represents a mean across the fifteen network-level values.

8 Investigating the mechanism for transmission inhibition

In this section we elaborate the statistical analyses used to test the predictions that networks that display only weak periodicity should also display weak transmission inhibition whereas periodic networks should exhibit stronger inhibition, and

To explore this prediction, we sought to test the null hypothesis that the magnitude of transmission inhibition did not depend upon the peak wavelet power. To do so, for each of the four transmission metrics, we performed a linear mixed-effects regression (Faraway, 2016) in which the response was the maximum absolute difference between the observed and expected transmission observed across all recovery rates (as shown by the y-position of the diamonds in Fig. 6 in the main paper), and the main effect, that is, the predictor of interest, was the dominant wavelet power (the y-position of the diamonds in Fig. 1h). In each mixed-effects model, colony identity (A,...,I), and replicate number (1,2) were also included as categorical random effects, with replicate number nested within colony identity. As the parameter-space exploration meant that the signal loss rate μ at which transmission was most inhibited could be estimated *for each level* of the transmission rate β, the transmission rate was also included as another random effect (0.01,...,0.1,...,1,...,10,...100).

All mixed-effects regressions were implemented using the *lme4* version 1.1-12 (Bates et al., 2015) for *R* version 3.3.2 (R Core Team, 2016). The statsitical significance of the main effects in the mixed effects models were calculated using *lmeTest* version 2.0-32 (Kuznetsova et al., 2015) for *R*. In order to conform to the LME model assumptions of residual normality, Yeo-Johnson transformations were applied to all four response variables (Yeo and Johnson, 2000). Example fits for a fixed value of the transmission rate, β=1, are shown in Figure S5a-c, and the overall summary statistics for each of the three models are given in Table S2.

Table S2: **Summary statistics for the mixed-effects regression models described in the main text**. The 'Y-J power' column refers to the Yeo-Johnson power transform that was applied to the response variable in order to obtain a mixed-effects model with normally distributed residuals.

Response	Y-J power	Main effect	Coefficient, β	SE(β)	d.f.	t	p
$min(P^{O-E}_{breakout})$	13	Peak wavelet power	-0.2	0.082	13	-2.4	0.033
$min(P^{O-E}_{sustain})$	21	Peak wavelet power	-0.13	0.041	10	-3.1	0.011
$min(P^{O-E}_{informed})$	4.7	Peak wavelet power	-0.39	0.17	13	-2.3	0.038
$max(P^{O-E}_{lost})$	-1.1	Peak wavelet power	1.6	0.44	9.8	3.5	$<$0.00001

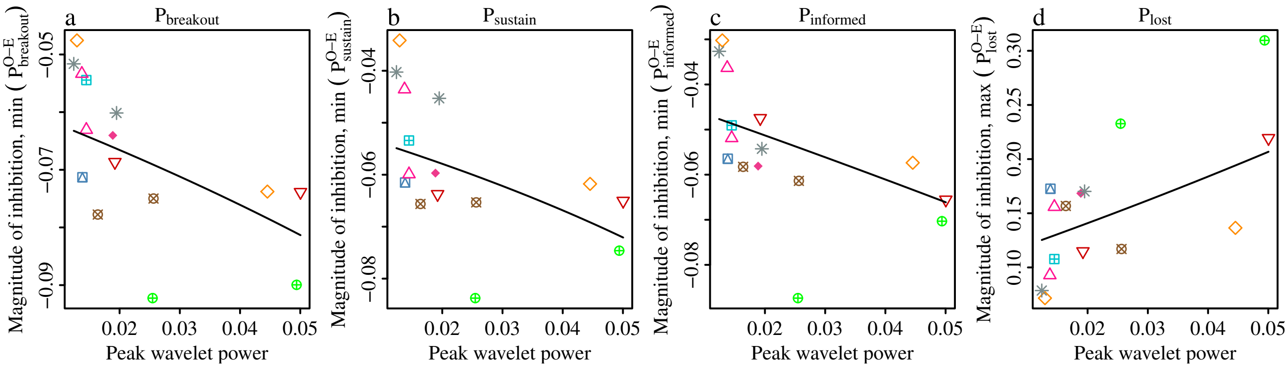

Figure S5: **Mixed-effects regression modelling supports the proposal that transmission inhibition is dependent upon activity cycle strength.** Each point represents a single 3-day contact network (N=15). Point types correspond to colony identity (A,...,I). As the transmission rate β was coded as a random effect in the LMER models, and as there were 40 different levels of β, for clarity we only show the fits for a single level (β=1). Note, the fits are non-linear because the response variables were subjected to Yeo-Johnson transformation.

References

Bates, D., M. Mächler, B. Bolker, and S. Walker (2015). Fitting linear mixed-effects models using lme4. *Journal of Statistical Software 67*(1), 1–48.

Boi, S., I. Couzin, N. Del Buono, N. Franks, and N. Britton (1999). Coupled oscillators and activity waves in ant colonies. *Proceedings of the Royal Society of London B: Biological Sciences 266*(1417), 371–378.

Briscoe, A. D. and L. Chittka (2001). The evolution of color vision in insects. *Annu. Rev. Entomol. 46*(1), 471–510.

Cazelles, B., M. Chavez, G. C. de Magny, J.-F. Guégan, and S. Hales (2007). Time-dependent spectral analysis of epidemiological time-series with wavelets. *Journal of the Royal Society Interface 4*(15), 625–636.

Cole, B. J. (1991). Short-term activity cycles in ants: generation of periodicity by worker interaction. *American Naturalist*, 244–259.

Cole, B. J. and D. Cheshire (1996). Mobile cellular automata models of ant behavior: movement activity of *Leptothorax allardycei*. *American Naturalist*, 1–15.

Cole, B. J. and L. Hoeg (1996). The influence of brood type on activity cycles *Leptothorax allardycei* (hymenoptera: Faruicidae). *Journal of insect Behavior 9*(4), 539–547.

Faraway, J. J. (2016). *Extending the linear model with R: generalized linear, mixed effects and nonparametric regression models*, Volume 124. CRC press.

Gibson, M. A. and J. Bruck (2000). Efficient exact stochastic simulation of chemical systems with many species and many channels. *The journal of physical chemistry A 104*(9), 1876–1889.

Holme, P. and J. Saramäki (2012). Temporal networks. *Physics Reports 519*(3), 97–125.

Isella, L., J. Stehlé, A. Barrat, C. Cattuto, J. Pinton, and W. Van den Broeck (2011). What's in a crowd? analysis of face-to-face behavioral networks. *Journal of Theoretical Biology 271*(1), 166–180.

Karsai, M., M. Kivelä, R. Pan, K. Kaski, J. Kertész, A.-L. Barabási, and J. Saramäki (2011). Small but slow world: How network topology and burstiness slow down spreading. *Physical Review E 83*, 025102(R).

Kuznetsova, A., P. B. Brockhoff, and R. H. B. Christensen (2015). Package 'lmertest'. *R package version 2*.

Leventhal, G. E., A. L. Hill, M. A. Nowak, and S. Bonhoeffer (2015). Evolution and emergence of infectious diseases in theoretical and real-world networks. *Nature Communications 6*, 6101.

Mersch, D. P., A. Crespi, and L. Keller (2013). Tracking individuals shows spatial fidelity is a key regulator of ant social organization. *Science 340*(6136), 1090–1093.

Newman, M. E. (2002). Spread of epidemic disease on networks. *Physical review E 66*(1), 016128.

Percival, D. B. and A. T. Walden (2006). *Wavelet methods for time series analysis*, Volume 4. Cambridge university press.

Perotti, J. I., H.-H. Jo, P. Holme, and J. Saramäki (2014). Temporal network sparsity and the slowing down of spreading. *arXiv preprint arXiv:1411.5553*.

R Core Team (2016). *R: A Language and Environment for Statistical Computing*. Vienna, Austria: R Foundation for Statistical Computing.

Roesch, A. and H. Schmidbauer (2014). *WaveletComp: Computational Wavelet Analysis*. R package version 1.0.

Yeo, I.-K. and R. A. Johnson (2000). A new family of power transformations to improve normality or symmetry. *Biometrika 87*(4), 954–959.
